# Association analysis between the TLR9 gene polymorphism rs352140 and type 1 diabetes

**DOI:** 10.3389/fendo.2023.1030736

**Published:** 2023-04-17

**Authors:** Yimeng Wang, Ying Xia, Yan Chen, Linling Xu, Xiaoxiao Sun, Jiaqi Li, Gan Huang, Xia Li, Zhiguo Xie, Zhiguang Zhou

**Affiliations:** National Clinical Research Center for Metabolic Diseases, Key Laboratory of Diabetes Immunology (Central South University), Ministry of Education, and Department of Metabolism and Endocrinology, The Second Xiangya Hospital of Central South University, Changsha, Hunan, China

**Keywords:** type 1 diabetes, autoimmunity, toll-like receptor, polymorphism, immunotherapy

## Abstract

**Background:**

To a great extent, genetic factors contribute to the susceptibility to type 1 diabetes (T1D) development, and by triggering immune imbalance, Toll-like receptor (TLR) 9 is involved in the development of T1D. However, there is a lack of evidence supporting a genetic association between polymorphisms in the TLR9 gene and T1D.

**Methods:**

In total, 1513 individuals, including T1D patients (n=738) and healthy control individuals (n=775), from the Han Chinese population were recruited for an association analysis of the rs352140 polymorphism of the TLR9 gene and T1D. rs352140 was genotyped by MassARRAY. The allele and genotype distributions of rs352140 in the T1D and healthy groups and those in different T1D subgroups were analyzed by the chi-squared test and binary logistic regression model. The chi-square test and Kruskal−Wallis H test were performed to explore the association between genotype and phenotype in T1D patients.

**Results:**

The allele and genotype distributions of rs352140 were significantly different in T1D patients and healthy control individuals (*p*=0.019, *p*=0.035). Specifically, the T allele and TT genotype of rs352140 conferred a higher risk of T1D (OR=1.194, 95% CI=1.029-1.385, *p*=0.019, OR=1.535, 95% CI=1.108-2.126, *p*=0.010). The allele and genotype distributions of rs352140 were not significantly different between childhood-onset and adult-onset T1D and between T1D with a single islet autoantibody and T1D with multiple islet autoantibodies (*p*=0.603, *p*=0.743). rs352140 was associated with T1D susceptibility according to the recessive and additive models (*p*=0.015, *p*=0.019) but was not associated with T1D susceptibility in the dominant and overdominant models (*p*=0.117, *p*=0.928). Moreover, genotype-phenotype association analysis showed that the TT genotype of rs352140 was associated with higher fasting C-peptide levels (*p*=0.017).

**Conclusion:**

In the Han Chinese population, the TLR9 polymorphism rs352140 is associated with T1D and is a risk factor for susceptibility to T1D.

## 1 Introduction

Characterized by absolute insulin deficiency and hyperglycemia, type 1 diabetes (T1D) is a chronic organ-specific autoimmune disease caused by defects in immune tolerance. Both the innate and adaptive immune systems, including T cells, B cells and dendritic cells, are involved in the destruction of β cells (1). Although the pathogenesis of T1D remains unclear, it is well recognized that a complex interaction between genetic and environmental factors leads to its occurrence and development (2). Indeed, genetic factors are highly related to T1D susceptibility. Extensive genetic studies have identified human leukocyte antigen (HLA) and more than 75 non-HLA gene loci as susceptibility genes related to T1D (3). HLA genes are responsible for nearly 50% of T1D risk, and HLA class II genes are the most significant determinants of T1D genetic susceptibility (4–6). Additionally, non-HLA genes contributing to T1D risk are predominantly involved in immune tolerance, immune function and β- cell function; these genes include insulin, protein tyrosine phosphatase nonreceptor type 22 (PTPN22), cytotoxic T-lymphocyte associated antigen 4 (CTLA4), and interleukin 2 receptor alpha (IL2RA), among others (2, 7).

Toll-like receptors (TLRs) are the most studied pattern recognition receptors and play an essential role in activating the innate immune response and regulating adaptive immune responses (8). There are 10 TLR subtypes (TLR1-10) expressed in humans (9). After TLRs recognize pathogen-associated molecular patterns, intracellular signaling pathways are activated to promote the release of proinflammatory cytokines and chemokines and upregulate the expression of costimulatory molecules (10). Therefore, aberrant activation of TLRs may participate in the development of autoimmune disorders by inducing proinflammatory pathways.

Numerous studies have suggested the involvement of many TLR subtypes in the pathophysiology of T1D, especially TLR9 (11). TLR9 is predominantly expressed in plasmacytoid dendritic cells (12) and is also present in B cells (13), T cells (14), epithelial cells (15), pancreatic ductal and endothelial cells (16). It has been reported that TLR9 loss results in a significantly decreased incidence of T1D in NOD mice, which is a classic mouse model for T1D research. These findings demonstrate that TLR9 functions as a critical risk factor for T1D. In NOD mice with TLR9 knockout, the frequencies of plasmacytoid dendritic cells and diabetogenic CD8+ T cells show a decreasing trend accompanied by lower levels of IFN-α in pancreatic lymph nodes, which may inhibit the development of T1D (17). Moreover, TLR9 deficiency in NOD mice markedly upregulates the expression of CD73 and enhances the immunosuppressive function of CD73+ T cells. Thus, the levels of proinflammatory cytokines are decreased, the expression levels of more anti-inflammatory cytokines are increased, and diabetes development is delayed (18). TLR9 mediates the regulation of interleukin (IL)-10- producing B cells, and the specific deletion of TLR9 in B cells alters the proportion and function of IL-10-producing B cells, downregulates their responsiveness to immune stimuli and suppresses T-cell-mediated responses. Through this mechanism, TLR9 deficiency in B cells can promote immune tolerance and strikingly protect NOD mice from developing T1D (19). Therefore, TLR9 deficiency has a protective role against T1D development and may be an effective therapeutic target.

The TLR9 gene is located at human chromosome 3p21.2. The relationship between TLR9 gene polymorphisms and autoimmune diseases has been reported. For example, the rs352140, rs187084 and rs5743836 polymorphisms of the TLR9 gene have been reported to be highly associated with a greater risk of developing systemic lupus erythematosus (SLE). In the Chinese population, significant differences in frequency distributions between SLE patients and control groups have been found for rs352140 (20, 21). Another study revealed a higher level of TLR9 transcripts in lupus nephritis biopsies and a significant genotypic and allelic association between rs352140 and both SLE and lupus nephritis (22). As a result of genetic adaptation to prevailing endemic infection, the more frequent T alleles of rs187084 and rs5743836 increase the expression of TLR9 and confer a significant risk for developing SLE in the Indian population (23, 24). In patients with rheumatoid arthritis (RA), an increase in TLR9 levels was detected in monocyte subgroups of the circulatory system and synovium (25). The rs352139 polymorphism of TLR9 has been associated with therapeutic efficacy in RA patients taking TNF−α inhibitors (26). Furthermore, the T allele of rs352140 is associated with higher susceptibility to ophthalmopathy in Chinese patients with Grave’s disease (GD) than the C allele of the rs352140 polymorphism (27). However, the relationship between TLR9 polymorphisms and T1D has not been studied, although existing evidence suggests its vital role in T1D development.

As mentioned above, the TLR9 rs352140 polymorphism was reported to be associated with susceptibility to other autoimmune diseases, including SLE and GD, in the Chinese population (20, 21, 27). Therefore, we explored the role of the TLR9 gene polymorphism rs352140 in T1D susceptibility in the Han Chinese population. The present study was the first to find the significant polymorphism in the TLR9 gene associated with T1D, aiming to provide evidence for the genetic association between TLR9 polymorphisms and the risk of T1D and a molecular indicator for the risk assessment, precise diagnosis and treatment of T1D.

## 2 Materials and methods

### 2.1 Study population

The study was approved by the Ethics Committee of Second Xiangya Hospital and carried out according to Helsinki Declaration guidelines. A total of 1513 Han Chinese individuals, including T1D patients (n=738) and healthy control individuals (n=775), were recruited. The T1D patients conformed to the following inclusion criteria: (1) meeting the criteria for diagnosing diabetes formulated by the WHO in 1999; (2) acute onset and diabetic ketosis or ketoacidosis under no obvious inducement within 6 months; (3) absolute insulin deficiency and relying on exogenous insulin within 6 months of diagnosis; (4) at least one kind of positive autoantibody targeting islets in the serum, including glutamic acid decarboxylase antibody (GADA), protein tyrosine phosphatase antibody (IA2A), and zinc transporter 8 antibody (ZnT8A); and (5) no pregnancy, malignant tumors, other types of diabetes or autoimmune diseases. The T1D patients were further categorized into different subgroups. Based on the age of disease onset, the 738 T1D patients were categorized as follows: 277 were childhood-onset patients (<18) and 461 were adult-onset patients (≥18). Based on the number of autoantibodies, the 738 T1D patients were categorized as follows: 383 patients were positive for a single islet autoantibody and 355 patients were positive for two or three islet autoantibodies.

Unrelated healthy individuals were enrolled as healthy control individuals according to the following criteria: (1) normal fasting plasma glucose (FPG) (<5.6 mmol/L) and 2-h postprandial plasma glucose (PPG) (<7.8 mmol/L) after a 75-g oral glucose challenge; (2) no family history of diabetes; and (3) no pregnancy, malignant tumors, chronic diseases or endocrine system diseases.

### 2.2 Collection of clinical measurements

The general information of the study subjects, including sex, age, natural course of T1D and treatment process, was collected by physicians. Anthropometric measurements, including weight and height, were also determined by physicians and used to calculate body mass index (BMI). Key biochemical measurements, including FPG, PPG, and fasting C-peptide, postprandial C-peptide and glycosylated hemoglobin (HbA1c) levels, were performed by technicians in the affiliated laboratories of the department. C-peptide levels and HbA1c levels were measured with a chemiluminescence method (ADVIA Centaur XP Immunoassay System, Siemens, Germany) and automated liquid chromatography (HLC-723G8, Tosoh, Japan), respectively; islet-specific autoantibodies were detected through a radioligand binding assay.

### 2.3 Blood collection and DNA extraction

Approximately 4 mL of peripheral blood was obtained from each individual. After collection, the blood samples were stored at -80 °C until DNA extraction or used immediately to extract DNA using a Genenode Genomic DNA Extraction kit (Genenode Biotech Co., Ltd., Beijing, China) according to the manufacturer’s instructions. The concentration and purity of the DNA were estimated using a NanoDrop ND-1000 spectrophotometer (Thermo Fisher Scientific, Waltham, MA, USA). DNA samples of acceptable quality were stored at -80 °C for further analysis.

### 2.4 SNP selection and genotyping

The single-nucleotide polymorphism (SNP) rs352140 in the TLR9 gene was screened for the association analysis based on the following principles: (1) minor allele frequencies in the Asian population >0.05; (2) *p* value of Hardy–Weinberg equilibrium >0.05; and (3) reported to be highly associated with the susceptibility to other autoimmune diseases, including SLE and GD, in the Chinese population. The SNP genotypes of individuals were identified by BGI (Beijing Genomics Institute, Shenzhen, China) through the MassARRAY^®^ Analyzer 4 System (Agena Bioscience, San Diego, CA, USA). The following primers were used for amplification in the study: forward, 5’-ACGTTGGATGTGGCTGTTGTAGCTGAGGTC-3’; reverse, 5’-ACGTTGGATGATAAGCTGGACCTCTACCAC-3’. The single base extension primer was 5’-GTCCAGGGCCTCCAGTCG-3’.

### 2.5 Statistical analysis

IBM SPSS Statistics version 26.0 was employed to perform statistical analyses. Hardy–Weinberg equilibrium testing in the control group was performed by the chi-square goodness of fit test. Normality tests showed that the continuous variables in the study did not follow a normal distribution (*p*<0.05). All continuous variables are presented as the median (interquartile range), and differences between the cases and control individuals were analyzed by the Mann−Whitney U test. Categorical data are expressed as absolute numbers and constituent ratios, and differences between the cases and control individuals were analyzed by the chi-squared test. The allele and genotype frequency distributions between the T1D and healthy individuals and between T1D subgroups were assessed by the chi-squared test. Logistic regression analysis and the chi-squared test were applied to further compare differences in the genotype frequency distributions between the two comparison groups under four classic genetic models. The odds ratio (OR) and 95% confidence interval (CI) were used to evaluate the association between the TLR9 polymorphism rs352140 and T1D susceptibility. Comparison of clinical characteristics was performed by the chi-square test and Kruskal−Wallis H test to explore the effects of different SNP genotypes on phenotypes in T1D patients. *p* < 0.05 was defined as statistically significant.

## 3 Results

### 3.1 General clinical characteristics of case and control subjects

A total of 738 T1D patients and 775 sex-matched healthy control individuals from the Han Chinese population were recruited for this study (403/335 vs. 415/360, *p*=0.680). The general information and biochemical measurements of the individuals are listed in **Table 1**. Significant differences between the T1D patients and control individuals were found for age and BMI (*p*<0.001, *p*<0.001). Compared with healthy control individuals, the T1D patients were younger and leaner. Significant differences in FPG and PPG were also observed (*p*<0.001, *p*<0.001). The blood glucose levels of the T1D patients were significantly higher than those of the control individuals.

**Table 1:** Clinical characteristics of the study subjects.

Characteristics	T1D group	Control group	*p* value
Sample size	738	775	–
Sex (men/women)	403/335	415/360	0.680
Age (year)	26 (14-37)	39 (25-49)	<0.001*
BMI (kg/m^2^)	19.14 (17.09-21.41)	22.50 (20.20-25.56)	<0.001*
T1D duration (month)	2.00 (0.30-12.00)	–	–
FPG (mmol/L)	8.40 (6.10-12.88)	5.10 (4.69-5.40)	<0.001*
PPG (mmol/L)	15.48 (10.92-20.45)	5.80 (4.90-6.60)	<0.001*
HbA1c (%)	10.50 (7.90-13.00)	–	–
FCP (pmol/L)	88.05 (32.34-170.13)	–	–
PCP (pmol/L)	177.50 (58.70-393.95)	–	–
GADA+	678 (91.87)	–	–
IA-2A+	365 (49.46)	–	–
ZnT8A+	251 (34.01)	–	–
Single Ab+	383 (51.90)		
Multiple Ab+	355 (48.10)		
GADA titer (U/mL)	392.30 (106.99-851.18)		
IA-2A titer (U/mL)	304.82 (75.21-871.52)		

BMI, body mass index; FPG, fasting plasma glucose; PPG, 2 h postprandial plasma glucose; HbA1c, glycated hemoglobin; FCP, fasting C-peptide; PCP, 2 h postprandial C-peptide; GADA, glutamic acid decarboxylase antibody; IA2A, protein tyrosine phosphatase antibody; ZnT8A, zinc transporter 8 antibody. Single Ab+, positive for a single islet autoantibody; Multiple Ab+, positive for two or three islet autoantibodies.The symbol “-” stands for “not applicable”.

### 3.2 Hardy-Weinberg equilibrium testing

The genotype frequency distributions of rs352140 in the control group were consistent with Hardy-Weinberg equilibrium (*p*=0.268); thus, the samples in the study were in genetic equilibrium and representative. More details are shown in **Table S1**.

#### 3.3 Distribution characteristics of the alleles and genotypes of rs352140

The genotypic frequency and allelic frequency of rs352140 were significantly different between the T1D and control groups (χ^2^= 6.681, *p*=0.035, *p*=0.019) (**Table 2**). For rs352140, the frequencies of the TT, TC and CC genotypes in the T1D individuals were 14.9%, 46.5% and 38.6%, respectively, and those in the healthy control individuals were 10.7%, 46.7% and 42.6%, respectively. The T and C allele frequencies were 38.1% and 61.9% in the T1D patients and 34.1% and 65.9% in the control individuals, respectively. The T allele and TT genotype frequency were significantly different between the T1D and control groups, indicating that the T allele and TT homozygote genotype conferred a higher genetic risk of developing T1D (OR=1.194, 95% CI=1.029-1.385, *p*=0.019, OR=1.535, 95% CI=1.108-2.126, *p*=0.010). However, there were no differences in the frequency distributions of the rs352140 allele and genotype between childhood-onset and adult-onset T1D patients (*p*=0.603) or between T1D patients positive for a single islet autoantibody and those positive for multiple islet autoantibodies (*p*=0.743) (**Table 3**).

**Table 2:** Association analysis between the rs352140 polymorphism and T1D risk.

rs352140	T1D group (n=738)	Control group (n=775)	OR (95% CI)	*p*
Genotype				0.035^*^
TT	110 (14.9)	83 (10.7)	1.535 (1.108-2.126)	0.010^*^
TC	343 (46.5)	362 (46.7)	1.097 (0.883-1.363)	0.402
CC	285 (38.6)	330 (42.6)	1	–
Allele				
T	563 (38.1)	528 (34.1)	1.194 (1.029-1.385)	0.019^*^
C	913 (61.9)	1022 (65.9)	1	–
Genetic models				
Dominant model				
TT + TC	453 (61.4)	445 (57.4)	1.179 (0.960-1.448)	0.117
CC	285 (38.6)	330 (42.6)	1	
Recessive model				
TT	110 (14.9)	83 (10.7)	1.460 (1.077-1.981)	0.015^*^
TC + CC	628 (85.1)	692 (89.3)	1	
Overdominant model				
TC	343 (46.5)	362 (46.7)	0.991 (0.809-1.213)	0.928
TT + CC	395 (53.5)	413 (53.3)	1	
Additive model				
TT	110 (14.9)	83 (10.7)	1.196 (1.030-1.389)	0.019^*^
TC	343 (46.5)	362 (46.7)		
CC	285 (38.6)	330 (42.6)		

The symbol “-” stands for “not applicable”.

**Table 3:** The analysis of the rs352140 polymorphism in T1D subgroups.

rs352140	T1D subgroups based on onset age				T1D subgroups based on autoantibody number			
	Childhood-onset (n = 277)	Adult-onset (n = 461)	OR (95% CI)	*p*	Single Ab+ (n = 383)	Multiple Ab+ (n = 355)	OR (95% CI)	*p*
Genotype				0.603				0.743
TT	46 (16.6)	64 (13.9)	1.232 (0.787-1.930)	0.362	54 (14.1)	56 (15.8)	0.844 (0.543-1.311)	0.449
TC	126 (45.5)	217 (47.1)	0.995 (0.719-1.379)	0.978	177 (46.2)	166 (46.8)	0.933 (0.681-1.278)	0.666
CC	105 (37.9)	180 (39.0)	1	–	152 (39.7)	133 (37.5)	1	–
Allele								
T	218 (39.4)	345 (37.4)	1.085 (0.874-1.347)	0.460	285 (37.2)	278 (39.2)	0.921 (0.746-1.136)	0.441
C	336 (60.6)	577 (62.6)	1	–	481 (62.8)	432 (60.8)	1	–
Genetic models								
Dominant model								
TT + TC	172 (62.1)	281 (61.0)	1.049 (0.772-1.426)	0.758	231 (60.3)	222 (62.5)	0.910 (0.677-1.225)	0.536
CC	105 (37.9)	180 (39.0)	1	–	152 (39.7)	133 (37.5)	1	–
Recessive model								
TT	46 (16.6)	64 (13.9)	1.235 (0.818-1.865)	0.315	54 (14.1)	56 (15.8)	0.876 (0.584-1.314)	0.523
TC + CC	231 (83.4)	397 (86.1)	1	–	329 (85.9)	299 (84.2)	1	–
Overdominant model								
TC	126 (45.5)	217 (47.1)	0.938 (0.696-1.265)	0.676	177 (46.2)	166 (46.8)	0.978 (0.732-1.307)	0.882
TT + CC	151 (54.5)	244 (52.9)	1	–	206 (53.8)	189 (53.2)	1	–
Additive model								
TT	46 (16.6)	64 (13.9)	1.084 (0.874-1.344)	0.463	54 (14.1)	56 (15.8)	0.922 (0.748-1.136)	0.445
TC	126 (45.5)	217 (47.1)			177 (46.2)	166 (46.8)		
CC	105 (37.9)	180 (39.0)			152 (39.7)	133 (37.5)		

The symbol “-” stands for “not applicable”.

### 3.4 Association analysis between rs352140 and T1D susceptibility in genetic models

Four classic genetic models were adopted to further explore the role of the rs352140 polymorphism in T1D susceptibility (**Table 2**). The TT genotype of rs352140 was related to a higher risk of T1D susceptibility under a recessive model (OR=1.460, 95% CI=1.077-1.981, *p*=0.015). Under an additive model, the frequencies of the rs352140 genotype were significantly different between the T1D and control groups (*p*=0.019). However, no significant associations between the rs352140 genotype and T1D susceptibility were observed under the dominant and overdominant models (*p*=0.117, *p*=0.928).

### 3.5 Association analysis between rs352140 and clinical characteristics of T1D

To evaluate a genotype-phenotype association in the development of T1D, we compared the clinical parameters of T1D individuals with different genotypes of rs352140, including homozygotes and heterozygotes (**Table 4**). The results showed that rs352140 was associated with residual islet β-cell function. Specifically, T1D patients with TT homozygosity showed higher FCP levels than patients with CC homozygosity (*p*=0.017). Other clinical characteristics were not significantly different among T1D patients with different genotypes of rs352140, including sex, age of onset, T1D duration, HbA1c, autoantibody positivity rate and titer (**Table 4**).

**Table 4:** Clinical characteristics of T1D patients with different rs352140 genotypes.

Clinical characteristics	Genotype			*p* value
	TT	TC	CC	
Sample size	110	343	285	–
Sex (men/women)	61/49	183/160	159/126	0.815
BMI (kg/m^2^)	19.20 (17.34-21.40)	19.10 (17.07-21.24)	19.12 (16.99-21.50)	0.867
Onset age (years)	22 (12-33)	25 (12-36)	23 (13-35)	0.631
T1D duration (months)	1.66 (0.24-12.50)	2.00 (0.30-11.88)	3.00 (0.36-13.00)	0.322
FCP (pmol/L)	113.10 (39.70-209.60)	102.00 (33.00-181.64)	66.66 (28.18-146.03)	0.017^*^
PCP (pmol/L)	207.50 (85.90-473.70)	189.25 (68.28-370.53)	143.26 (40.25-384.83)	0.099
HbA1c (%)	11.00 (7.90-13.15)	10.20 (7.8-12.90)	10.45 (8.20-13.20)	0.565
GADA+	102 (92.73)	322 (93.88)	254 (89.12)	0.089
IA-2A+	56 (50.91)	169 (49.27)	140 (49.12)	0.946
ZnT8A+	42 (38.18)	113 (32.94)	96 (33.68)	0.595
GADA titer (U/mL)	410.97 (148.03-872.65)	392.30 (98.35-900.10)	332.06 (99.61-783.60)	0.367
IA-2A titer (U/mL)	394.47 (115.31-963.78)	264.16 (55.25-775.65)	290.46 (96.29-918.88)	0.169

*p<0.05.

## 4 Discussion

Genetic factors exert significant impacts on susceptibility to T1D. TLRs act as mediators of innate and adaptive immune responses by recognizing different kinds of molecular patterns and promoting the expression of proinflammatory cytokines and costimulatory molecules (8, 28, 29). However, the aberrant expression and activation of TLRs can cause immune imbalances and abnormal immune responses, thus leading to the development of immune-related diseases. Numerous studies have suggested the significant role of TLR9 in the pathogenesis of autoimmune diseases, including T1D (17–19). It has been reported that polymorphisms in the TLR9 gene are related to an increased risk of developing other autoimmune diseases, including SLE, RA and GD (20, 26, 27). However, polymorphisms in the TLR9 gene associated with the risk of T1D have not been reported.

The present study was designed to explore the association between the TLR9 polymorphism rs352140 and T1D susceptibility in the Han Chinese population. We enrolled 738 T1D patients and 775 healthy control individuals and extracted DNA from blood samples to analyze the distribution characteristics of rs352140. The results showed that the distribution frequencies of rs352140 were significantly different between the T1D patients and healthy control individuals. The T allele of rs352140 conferred a significant risk of developing T1D. We then analyzed the role of rs352140 in susceptibility to T1D by adopting four genetic models. rs352140 was found to be related to T1D susceptibility under both a recessive model and an additive model but not the dominant and overdominant models. These results indicate that the effect of one copy of the T allele is not enough to play a marked role in offsetting the effect of the C allele and that only two copies of the T allele can exhibit an effect on increasing the risk of developing T1D. Another possible explanation is that the sample size was not large enough to find an association between rs352140 and T1D susceptibility under the dominant and overdominant models. Finally, we further identified whether the genotypes of rs352140 affect the phenotypes of T1D patients. We found that T1D patients with the rs352140 TT genotype showed higher levels of fasting C-peptide, indicating that rs352140 is associated with residual β-cell function. These findings suggest that rs352140 is linked to genetic susceptibility to T1D, with a vital role in its pathogenesis.

rs352140, located in the second exon region of the TLR9 gene, is a synonymous variant. Although synonymous variants do not lead to amino acid alterations, they may also affect the splicing, stability and structure of mRNA and protein folding, thus influencing the structure, expression and function of proteins (30, 31). rs352140 is associated with higher TLR9 expression in patients with primary biliary cirrhosis, an autoimmune liver disease, and infected by hepatitis virus (32, 33). Another study showed that the significant genotypic and allelic association between rs352140 and lupus nephritis was accompanied by a higher level of TLR9 transcripts in biopsies of lupus nephritis patients (22). These results indicate that rs352140 may change the gene expression of TLR9, probably enhancing its expression. Therefore, rs352140 may be involved in the development of T1D by increasing the expression levels of TLR9 and then altering immune response patterns. Besides, the comprehensive analysis of genetics, transcriptomics, proteomics and epigenomics data shows that rs352140 may also affect the splicing and expression of other genes, such as GLYCTK, PPM1M and ITIH4 (Open Targets Genetics, http://genetics.opentargets.org).

Most notably, individuals from the control group were older than the T1D patients, which may be attributed to the peak incidence of T1D. Indeed, most epidemiological studies have shown that T1D is more likely to present in adolescence; hence, we chose older control individuals who had a lower possibility of developing T1D in the future (34). Confusingly, the TT genotype was related to an increased risk of T1D but was associated with improved β-cell function. ODN 1585, a TLR9 agonist, is reported to prolong pancreatic islet allograft survival and enhance insulin production by enhancing the expansion of the natural killer cell subsets producing IL-22 in the liver in T1D mice (35). However, another study showed that TLR9 deficiency can promote the development and differentiation of pancreatic islet β cells, enhancing glucose tolerance and improving insulin sensitivity in T1D mice. Treatment with a TLR9 antagonist can also improve β-cell function by increasing the number of islet β cells expressing CD140a as well as the number of β cells (36). Therefore, the relationship between TLR9 polymorphisms and β cell function needs to be further investigated in larger samples.

Genome wide association studies for T1D have been performed in the Caucasian populations, Chinese Han population and Japanese population (37–39). However, no SNPs in the TLR9 gene including rs352140 were identified in those studies. Another study showed that TLR9 SNP rs3796508 was not associated with the susceptibility of T1D (40). The association between other polymorphisms in the TLR9 gene and the risk of T1D has not been reported by other genetic researches. Therefore, this is the first study to find the significant polymorphism in the TLR9 gene associated with the risk of T1D. Although numerous studies have demonstrated that TLR9 has a significant role in the development of T1D, most work related to TLR9 has been performed in a T1D mouse model and not in T1D patients. Our study provides evidence for the significant role of TLR9 in the development of T1D in humans. Moreover, the TLR9 polymorphism in the present study has been identified to correlate with a higher risk of SLE and GD, indicating that rs352140 is essential for promoting autoimmunity.

Apart from TLR9, other TLR subtypes have been identified as participating in the development of T1D, including TLR2, TLR3, TLR4 and TLR7 (11). Several genetic studies have identified many T1D-related polymorphisms for other TLRs, including polymorphisms in the TLR1 (rs4833095 and rs5743612) (40), TLR2 (rs3804100) (41), and TLR3 (rs5743313, rs5743315, rs3775291 and rs13126816) genes (42, 43). Considering the key role of TLRs in autoimmunity, these receptors are emerging as important therapeutic targets for autoimmune diseases, and small molecule modulators targeting TLR signaling have attracted much attention (44, 45).

Nevertheless, there are several limitations. First, only one SNP was chosen to analyze the relationship between TLR9 polymorphisms and T1D. To better understand the role of TLR9 in the genetic predisposition toward T1D, it is necessary to screen for more TLR9 SNPs in different regions, including introns, exons, 3’ untranslated regions and 5’ untranslated regions, and to perform haplotype analysis of TLR9 SNPs in future research. Second, our study lacks mechanistic details, and such research is necessary to identify the specific function and signaling pathways mediated by TLR9 polymorphisms in the course of T1D. Finally, the results are limited to the Han Chinese population. Genetic susceptibility to T1D varies in different ethnic populations, and environmental factors collaborate with genetic factors to participate in the pathogenesis of T1D. Whether the results of this study are universal remains to be further assessed in a larger sample size and multiple ethnic populations.

In conclusion, our study shows that the rs352140 polymorphism of TLR9 is associated with T1D in the Han Chinese population, and a higher frequency of the T allele of rs352140 results in a greater risk of developing T1D. Our study provides evidence for the significant role of TLR9 in the genetic susceptibility to T1D, thus providing a promising target for the risk assessment, precise diagnosis and immunotherapy for T1D.

## Data availability statement

All datasets presented in this study are available in the article/**Supplementary Material**. Further inquiries can be directed to the corresponding author.

## Ethics statement

The studies involving human participants were reviewed and approved by the Ethics Committee of Second Xiangya Hospital. Written informed consent to participate in this study was provided by the participants or participants' legal guardian/next of kin.

## Author contributions

YW designed the experiments, analyzed the data and wrote the manuscript. YX, YC, LX, XS, JL, GH, XL and ZZ critically revised the manuscript and provided substantial scientific contributions. ZX contributed to the study design, proposed the project and revised the manuscript. All authors contributed to the article and approved the submitted version.
